# Supplementary material for: A Meta-Analysis of Bacterial Communities in Food Processing Facilities: Driving Forces for Assembly of Core and Accessory Microbiomes across Different Food Commodities
Source: Microorganisms. 2023 Jun 14;11(6):1575. doi: 10.3390/microorganisms11061575 (PMC10304558; doi:10.3390/microorganisms11061575)
Supplement: Supplementary file 1 [file microorganisms-11-01575-s001.zip › microorganisms-2418678-supplementary.pdf]

Online Supplementary Material to

A meta-analysis of bacterial communities in food processing facilities: Driving forces for assembly of core and accessory microbiomes across different food commodities

Zhaohui S. Xu, Tingting Ju, Xianqin Yang and Michael Gänzle

Figure S1. Principal coordinates analysis (PCoA) with Jaccard index for bacterial diversity based on nutrients intensity of environmental samples from food processing facilities.

Figure S2. Multiple correspondence analysis (MCA) plots for bacterial diversity based on nutrients intensity of environmental samples from food processing facilities.

Figure S3. Multiple correspondence analysis (MCA) plots for bacterial diversity among different type of processing facilities associated with different nutrients level.

Table S1. List of publications and samples used in this study (provided as excel file).

Table S2. Permutational multivariate analysis of variance on Jaccard distance matrix of samples from the 5 food commodities to test the association of community variance.

Table S3. Permutational multivariate analysis of variance on Jaccard distance matrix of samples from high-nutrient surfaces to test the association of community with different food commodity variables.

Table S4. Permutational multivariate analysis of variance on Jaccard distance matrix of samples from low-nutrient surfaces to test the association of community with different food commodity variables.

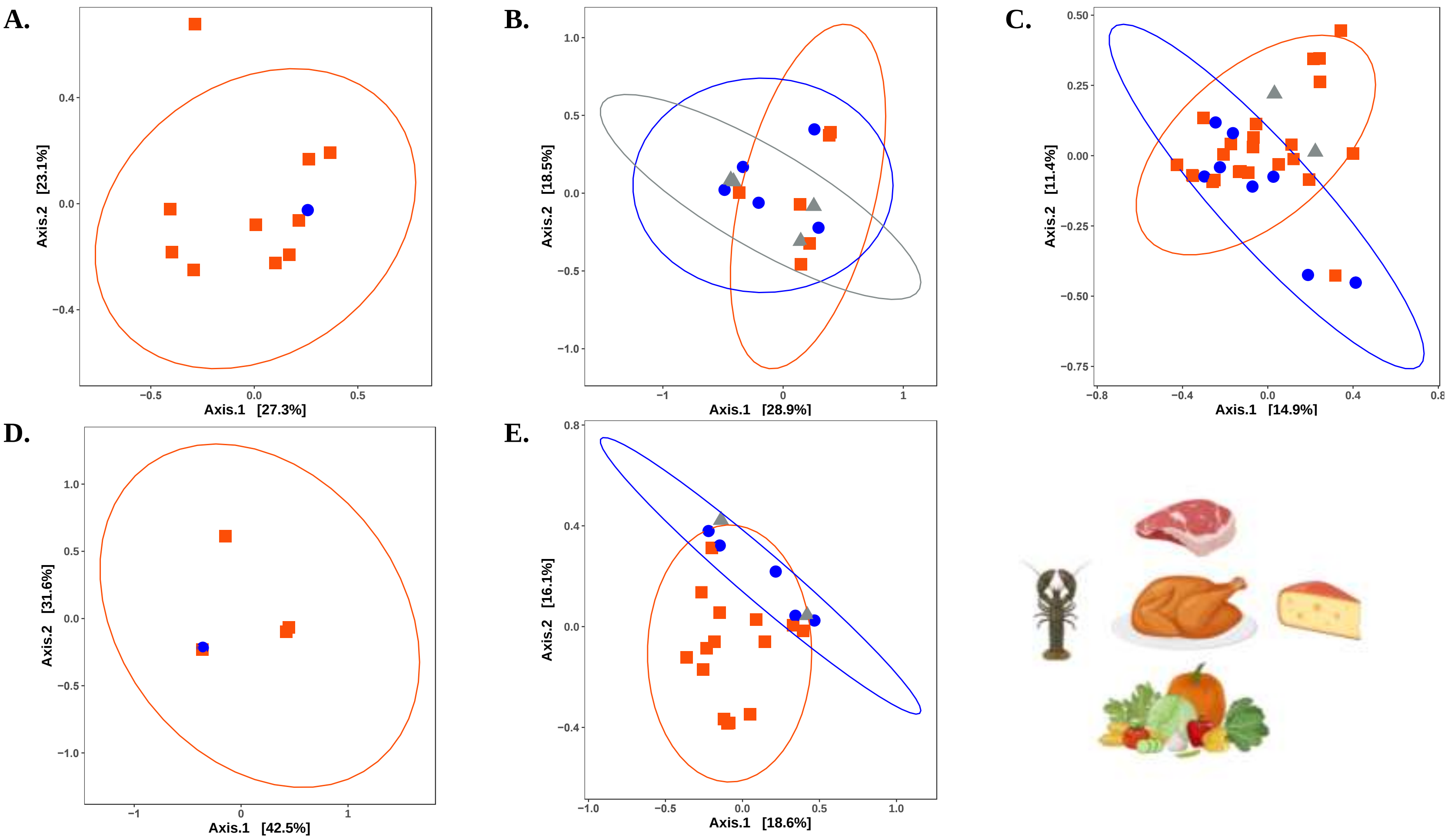

Figure S1. Principal coordinates analysis (PCoA) with Jaccard index for bacterial diversity based on nutrients intensity of environmental samples from food processing facilities. A, seafood processing facilities; B, fresh produce processing facilities; C, meat processing facilities; D, RTE processing facilities; E, cheese processing facilities. High nutrient (red); Low nutrient (blue); Unknown (grey). Permutational multivariate analysis of variance was used to statically differentiate bacterial communities. The composition of bacterial communities on low and high nutrient surfaces collected from cheese processing facilities differed significantly ($P < 0.05$). Statistical difference was not observed in other food commodities due to the small sample size of unknown and low nutrient surfaces.

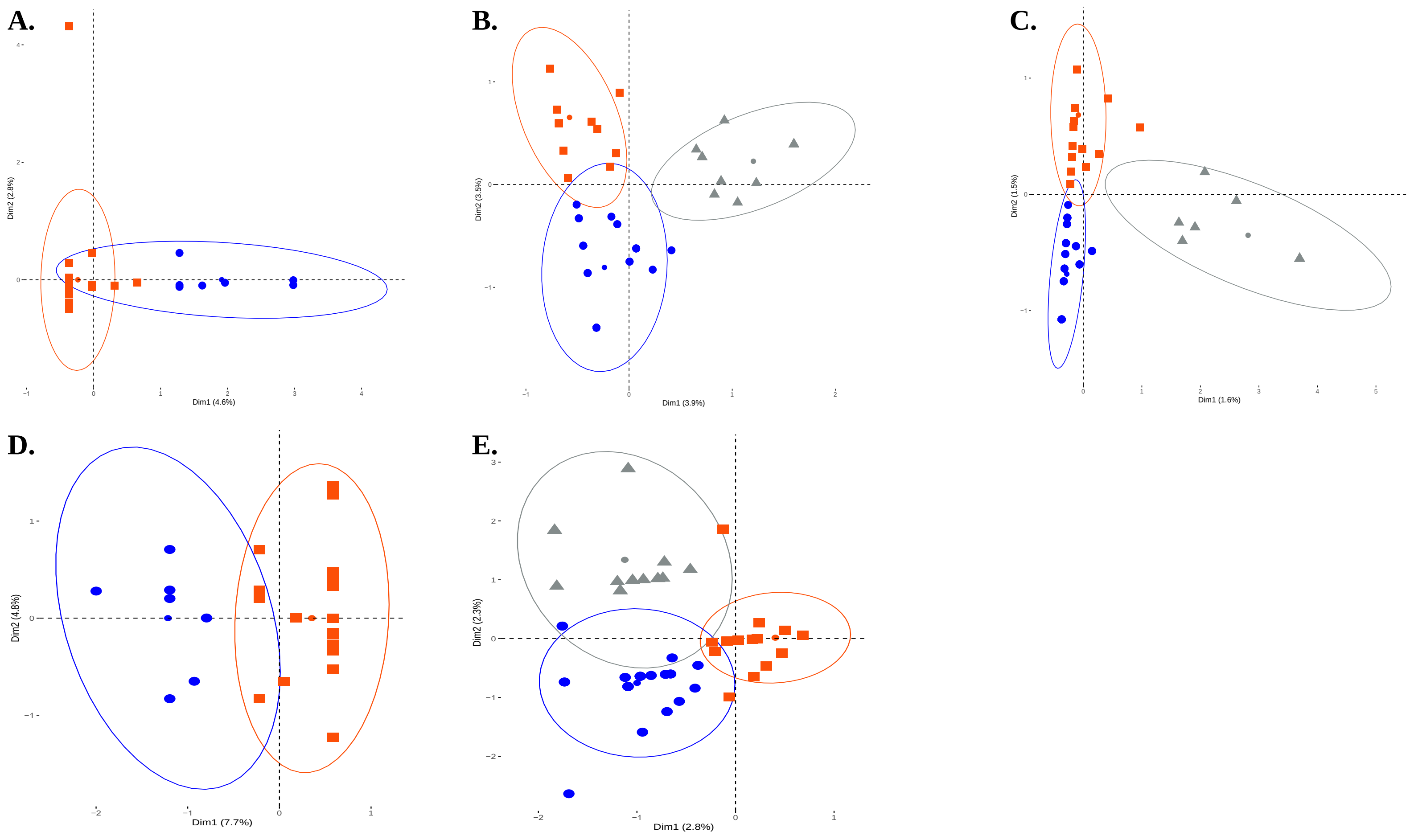

Figure S2. Multiple correspondence analysis (MCA) plots for bacterial diversity based on nutrients intensity of environmental samples from food processing facilities. A, seafood; B, fresh produce; C, meat; D, RTE; E, cheese. High nutrient (red); Low nutrient (blue); Unknown (grey).

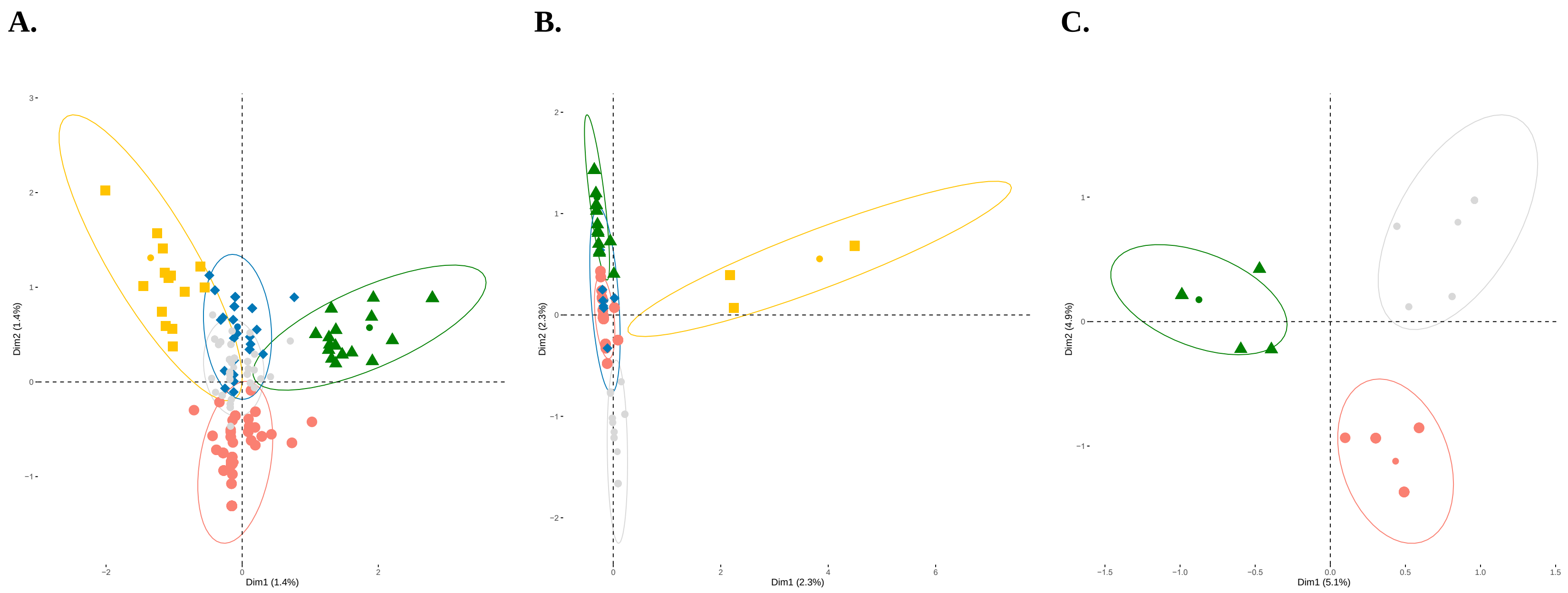

Figure S3. Multiple correspondence analysis (MCA) plots for bacterial diversity among different type of processing facilities associated with different nutrients level. Points represent data collected from different processing facilities and are clusters based on same nutrient level: A, high; B, low; C, unknown. Light grey, cheese processing facilities; green, fresh produce processing facilities; red, meat processing facilities; yellow, RTE processing facilities; blue, seafood processing facilities.

Table S2. Permutational multivariate analysis of variance on Jaccard distance matrix of samples from the 5 food commodities to test the association of community variance.

Comparison	P. value
Ready to eat vs Meat processing facility	0.47
Ready to eat vs Seafood processing facility	0.16
Ready to eat vs Fresh produce facility	0.18
Ready to eat vs cheese processing facility	0.09
Meat processing facility vs Seafood processing facility	0.01
Meat processing facility vs Fresh produce facility	0.01
Meat processing facility vs cheese processing facility	0.01
Seafood processing facility vs Fresh produce facility	0.01
Seafood processing facility vs cheese processing facility	0.01
Fresh produce facility vs cheese processing facility	0.01

Table S3. Permutational multivariate analysis of variance on Jaccard distance matrix of samples from high-nutrient surfaces to test the association of community with different food commodity variables.

**Comparison**	**P-value**
Ready to eat vs Meat processing facility	1.00
Ready to eat vs Seafood processing facility	1.00
Ready to eat vs Fresh produce facility	1.00
Ready to eat vs cheese processing facility	1.00
Meat processing facility vs Seafood processing facility	0.01
Meat processing facility vs Fresh produce facility	0.25
Meat processing facility vs cheese processing facility	0.05
Seafood processing facility vs Fresh produce facility	0.86
Seafood processing facility vs cheese processing facility	0.02
Fresh produce facility vs cheese processing facility	0.24

Table S4. Permutational multivariate analysis of variance on Jaccard distance matrix of samples from low-nutrient surfaces to test the association of community with different food commodity variables.

**Comparison**	**P-value**
Ready to eat vs Meat processing facility	1.00
Ready to eat vs Seafood processing facility	NA
Ready to eat vs Fresh produce facility	1.00
Ready to eat vs cheese processing facility	1.00
Meat processing facility vs Seafood processing facility	1.00
Meat processing facility vs Fresh produce facility	0.108
Meat processing facility vs cheese processing facility	0.018
Seafood processing facility vs Fresh produce facility	1.00
Seafood processing facility vs cheese processing facility	1.00
Fresh produce facility vs cheese processing facility	0.05
